# The influence of empathic concern on prosocial behavior in children

**DOI:** 10.3389/fpsyg.2014.00425

**Published:** 2014-05-12

**Authors:** Amanda Williams, Kelly O’Driscoll, Chris Moore

**Affiliations:** Department of Psychology and Neuroscience, Dalhousie UniversityHalifax, NS, Canada

**Keywords:** empathy, prosocial behavior, children

## Abstract

This research explored the influence of empathic distress on prosocial behavior in a resource allocation task with children. Children were randomly assigned to one of two conditions before engaging in a sticker sharing task; watching either a video of a girl upset that her dog had gone missing (emotion induction condition), or a video of the same girl preparing for a yard sale (control condition). In study one, 5–6 year old children in the emotion induction condition rated the emotional state of both the protagonist and the self more negatively, and also exhibited more prosocial behavior; sharing more in advantageous inequity (AI) trials, and less often withholding a benefit in disadvantageous inequity trials, than the control group. Prosocial behavior was significantly correlated with ratings of the emotional state of the protagonist but not with own emotional state, suggesting that empathic concern rather than personal distress was the primary influence on prosocial behavior. In study two, 3-year-olds were tested on AI trials alone, and like the 5 and 6-year-olds, showed more prosocial behavior in the emotion induction condition than the control.

## INTRODUCTION

It is well established that prosocial behavior such as helping and sharing emerges early in development (e.g., Rheingold et al., 1976; Warneken and Tomasello, 2006). A common approach to the study of sharing is to examine children’s resource allocation to self and others under various conditions. Preschool aged children will share valued resources and before long seek to establish fair allocations of resources across individuals (Thompson et al., 1997; Fehr et al., 2008; Brownell et al., 2009; Moore, 2009; Blake and McAuliffe, 2011). Although we know that preschool children will share, little is known about the mechanisms underlying such prosocial behavior (Hepach et al., 2013). By understanding these mechanisms, it should be possible to support and encourage the development of these highly valued, and critically important social behaviors.

Here we examine the role of empathic distress on young children’s decisions to allocate resources to another person. It is important to note that definitions of empathy in previous research have varied considerably across laboratories. Generally, however, empathy is believed to be a complex, and multifaceted construct consisting of a variety of components such as perspective taking, empathic concern, and personal distress (Davis, 1980). While empathic concern refers to the individual’s other oriented feelings of sympathy and concern for someone in distress, personal distress refers to experiencing unpleasant feelings oneself, in response to witnessing another in distress (Davis, 1980, 1983). In the context of this research, by empathic distress, we are referring to both personal distress and empathic concern. Our measure of personal distress is children’s own emotional reactions in response to a fictitious character’s situation (i.e., the tendency to experience the same negative emotion as another who is observed to be in distress). Our measure of empathic concern is children’s attributions of emotion to another who is observed to be in distress, without necessarily experiencing sadness them selves.

Empathy emerges early on, with infants exhibiting simple forms of global empathy by responding with reactive or contagious crying to observed distress in others (Sagi and Hoffman, 1976). At this young age, however, children lack the ability to differentiate between their own and others feelings (Hoffman, 1975, 1977). With time, however, children learn to distinguish and separate their own reactions from another individual’s distress. Around 2 years of age, children begin to develop the ability to understand the emotional states of others, experience and share their emotions, and make attempts to alleviate observed distress (Zahn-Waxler and Radke-Yarrow, 1990). As children continue to develop, they become increasingly sophisticated in their ability to understand and respond to the psychological states of others (Selman, 1980), and cultivate the ability to empathize with others in a more complex manner (Hoffman, 1975, 1977).

A large body of research has explored relationships between empathic distress, and various social behaviors or characteristics, and results have been mixed, varying in part according to how empathy and the behaviors or characteristics in question have been measured (Eisenberg and Miller, 1987). There is, however, evidence for a relation between empathic distress, or experiencing concern for others and prosocial behavior in children (e.g., Eisenberg et al., 1988; Zahn-Waxler and Radke-Yarrow, 1990; Strayer and Roberts, 1997; Malti et al., 2009; Vaish et al., 2009; see Eisenberg et al., 2006 for a review). For example, a relation has been found between children’s degree of facial sadness while watching a video of a child falling and hurting themselves, and later spontaneous sharing behavior with a partner (Eisenberg et al., 1988). In one study, empathy was found to be positively related to prosocial and social behaviors, and negatively associated with anger and aggression (Strayer and Roberts, 2004).

Batson et al. (1981) found that, in a sample of undergraduate students, helping behavior differed depending on the degree to which they experienced empathic concern, and the “ease of escape” or the cost to the subject for not helping the individual in distress. It was found that the helping behavior of participants high in empathic emotion was unaffected by ease of escape, suggesting their motivation was more purely altruistic and focused on alleviating the distress of the victim. In contrast, participants motivated to reduce their own distress were more likely to help when escape was difficult, and less likely to help when escape was easy. Similarly, with both adults and children, Eisenberg et al. (1989) also found that outward expressions of concern were positively related to prosociality, while personal distress was not.

In one of the few experimental studies exploring the effects of witnessing another individual in distress on prosocial behavior (Vaish et al., 2009), children were assigned to either a harm (witnessed one experimenter destroying or breaking something of value to another experimenter) or no harm condition (experimenter destroyed or broke an item not of value to the second experimenter). It was found that children in the harm condition exhibited more prosocial behavior toward the experimenter in a subsequent task, and that children’s facial concern in response to the experimenter in distress correlated with subsequent helping behavior, even without the experimenter exhibiting overt behavioral cues of distress. This research demonstrates that witnessing another individual in a distressing situation facilitates helping behavior in young children, and suggests that feeling concern for the distressed individual may be motivating this behavior.

With few exceptions (e.g., Vaish et al., 2009), much of the research in this area is correlational and such findings do not allow the conclusion that empathic distress leads to increased prosocial behavior. Also, as previously mentioned, research in the past has often not clearly differentiated between the effects of personal distress and empathic concern. A need exists, therefore, for experimental manipulation of emotional experience to examine, and distinguish between, the effects of personal distress and empathic concern on prosocial behavior in children. Further, although some research has explored how empathy is negatively related to anger or aggression (Strayer and Roberts, 2004) the literature focuses mainly on how empathy motivates positive facets of prosocial behavior. We were interested in exploring not only the positive effects of empathy on prosocial behavior in situations of advantageous inequity (AI) such as sharing (where the child can choose more resources for themselves, or to split resources equally between themselves and their partner), but also the potential mediating effects of empathy on potential non-prosocial behavior that is often observed in situations of disadvantageous inequity (DI; Fehr et al., 2008). In situations of DI, children must decide whether they would like to withhold resources from their partner to ensure they receive the same number of resources as themselves, or alternatively they can choose to deliver the extra resources to their partner. Within the literature, trials of DI have been referred to as “envy trials” (Fehr et al., 2008), with children who choose to withhold resources from their partner to prevent them from receiving more in DI trials believed to be exhibiting envious behavior. Envy is broadly conceptualized as a painful or resentful emotional experience associated with longing for, or wanting something that someone else has. Although the effects of empathy on envy have not been previously explored, one might predict that empathy could neutralize any negative or hostile emotions triggered in an inequitable context, thereby decreasing non-prosocial behavior, and encouraging prosocial behavior.

## STUDY 1

In this work, we adapted an approach previously used with adults to examine how induced emotion affects resource allocation. Barraza and Zak (2009) assigned participants to watch either a sadness inducing video of a father describing his experiences with his terminally ill son, or a neutral control video of a father and son at the zoo. Participants rated the degree to which they felt different emotions after watching the video, and then took part in an ultimatum game before being asked if they would like to donate their earnings to charity. Participants who watched the sadness inducing video later reported higher levels of negative emotion than those who watched the control video, which corresponded with more generous donations. Following this approach, we randomly assigned children to watch either a sadness inducing video of a young girl named Jenny upset that her dog had gone missing, or a neutral control video of the same girl preparing for a yard sale. Importantly, some potential limitations of the Barraza and Zak (2009) study were addressed by ensuring the videos were closely matched across conditions, inducing empathy for the recipient as opposed to an unrelated stranger, and exploring how empathy increased prosociality in a variety of resource allocation situations.

Our goal was to explore whether inducing a negative emotion, leading to empathetic distress, increases children’s prosocial behavior in a choice based resource allocation task. We asked children to rate their own emotion, as well as Jenny’s emotion to ensure that the emotion induction was inducing empathic distress, and also to explore if prosocial behavior was more strongly tied to either the empathic concern or personal distress aspect of empathy. The resource allocation task was chosen in order to explore the effects of empathic distress across both AI and DI decisions. Five and 6-year-old children participated in the resource allocation task, drawn from previous research by Fehr et al. (2008) and Moore (2009), which explored pre-school, and early school aged children’s behavior in sharing, prosocial, (AI) and envy (DI) trials. Over a series of four repetitions of four different trial types, children made decisions about how to allocate resources to themselves and a fictional partner (Jenny) by choosing one of two options. In each trial there was an equal option (participant and partner both received one sticker) and an unequal option. In AI trials (one with a cost, and one with no cost) the unequal option in both trial types benefited the participant alone, therefore the equal option was the prosocial choice. In contrast, in DI trials (again, with both a no cost and cost format) the unequal option delivered a greater benefit to Jenny, rendering the unequal option the prosocial choice. For the purpose of this study, therefore, envious behavior in DI trials was defined as making decisions in a way that prevents one’s partner from receiving a larger reward than the self, or withholding a benefit from one’s partner (e.g., when offered a choice between one sticker each or one for self and two for partner, the participant chooses the former option). By not exhibiting envy in DI trials, one would be exhibiting prosocial behavior. Our hypothesis was that children who were primed to feel empathy for their partner would be more likely to deliver a benefit to their partner in AI trials, and less often withhold a benefit from their partner in DI trials.

### METHOD

#### Participants

Fifty typically developing, 5 and 6-year old Canadian children drawn from a predominately white middle-class neighborhood participated in this study, which was approved by the University’s research ethics board. Children were randomly assigned to the emotion induction or control conditions, with 16 males, and 9 females in each group. The emotion induction group ranged in age from 61 months, 6 days, to 81 months, 29 days (*M* = 68 months, 24 days). The control group ranged in age from 60 months, 6 days, to 81 months, 25 days (*M* = 68 months, 26 days).

#### Emotion induction manipulation

Two videos were constructed for the purposes of this study. Both videos begin with a young girl, Jenny, playing in the backyard with her dog. In the emotion induction video, the dog runs away, and Jenny makes “lost dog” posters, which she hangs around her neighborhood. Jenny narrates in a sad tone and is visibly upset. In the control video Jenny is called inside, and makes and distributes “yard sale” posters for an upcoming yard sale while narrating in a neutral tone, and maintaining neutral facial expressions. The videos were matched on a number of pertinent factors: both were roughly 130 s in length, contained similar scenes and scene sequences, and were narrated according to scripts with almost identical structures and word counts. The prominent difference between the videos is the negative emotion displayed by the protagonist in the emotion induction video.

#### Procedure

Parental consent was obtained for each participant prior to testing. All children were tested in the laboratory in a session lasting roughly 20–25 min. The session included two phases: emotion induction followed by a resource-allocation task.

***Emotion induction***. Children sat in front of a 15-inch computer screen. The experimenter then briefly introduced the video’s content. Children were asked to focus on how Jenny felt, and how her story made them feel. They then watched the video.****

At the end of the video, children were asked to express how Jenny felt during the video. Children were then shown the Facial Affective Scale (FAS; McGrath et al., 1985 as cited in Perrott et al., 2004). The FAS is a 9-point measure that includes a range of happy and sad facial expressions, with a neutral face at its center point. Children were asked to point to a face that showed how they felt while viewing the video (emotion rating for self, providing a measure of personal distress) and a face that showed how they thought Jenny felt (emotion rating for Jenny, providing a measure of empathic concern). Potential scores on the FAS ranged from zero (happiest face) to eight (saddest face).

***Resource-allocation task***. This task adopted the method used by Fehr et al. (2008) and Moore (2009). The task consisted of 17 trials; one practice trial in which children could choose one or two stickers for themselves (demonstrating the format of the task), followed by four repetitions of each test trial, which offered the child a forced choice between two alterative distributions of stickers. AI and DI trials were blocked and counterbalanced with blocks separated by a distracter task (coloring a picture). In AI no cost trials, children chose between the allocation (1, 1) and (1, 0) – (one sticker for themselves and one for Jenny or one for themselves and none for Jenny). In AI cost trials, children chose between (1, 1) and (2, 0), in DI no cost trials, between (1, 1) and (1, 2), and in DI cost trials between (1, 1) and (2, 3). In all trials, the experimenter presented the choices by asking, “*Would you like one sticker for yourself and one sticker for Jenny or would you like {x} sticker(s) for yourself and {x} sticker(s) for Jenny?”* Upon completion, children in the emotion induction condition were told that Jenny’s dog returned home in order to neutralize any feelings of sadness.

### RESULTS

#### Manipulation check

**FIGURE 1:**
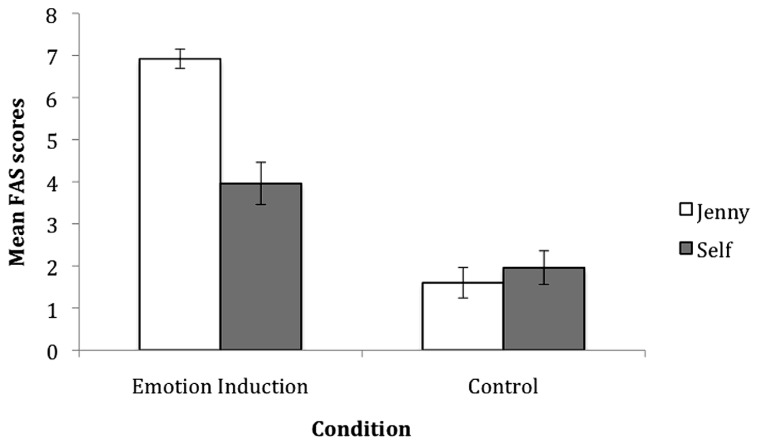
**Mean ratings for Jenny and self on the Facial Affective Scale (FAS), with standard error bars, for the emotion induction and control group in study one (5–6 year-olds).** Possible scores ranged from “0” (very happy) to “8” (very sad).

To ensure the emotion induction video was producing the desired effect, FAS scores for Jenny and self were compared across conditions (see **Figure 1** for mean scores). Independent samples *t*-tests showed that children in the emotion induction condition rated both Jenny’s and their own emotion as more negative than those in the control group (Jenny’s emotion, *t*(48) = 12.21, *p* < 0.01; own emotion *t*(48) = 3.11, *p* < 0.01). The mean score for Jenny’s emotion was 6.92 (SD = 1.18) in the emotion induction group and 1.6 (SD = 1.8) in the control group, while the mean score for own emotion was 3.96 (SD = 2.5) in the emotion induction group and 1.96 (SD = 2.0) in the control group. The differences between groups in both self-reported emotion, and perceptions of Jenny’s emotion show that the manipulation was successful, and empathy was induced by the emotion induction video. Further, a Pearson correlation between ratings for Jenny and self-showed a strong positive relationship, *r* = 0.529, *p* < 0.01, demonstrating that children who rated Jenny’s emotion as negative also rated their own emotion more negatively.

#### Main analysis

**FIGURE 2:**
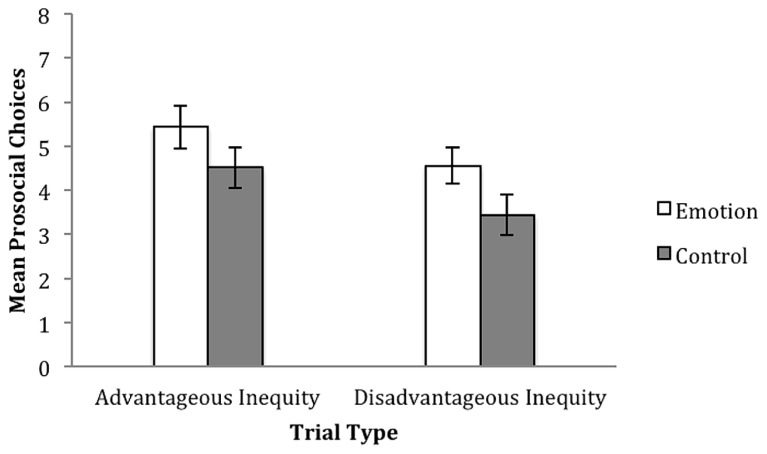
**Mean prosocial choices on the resource allocation task with standard error bars, for the emotion induction, and control group, in AI and DI trials in study one (5–6 year-olds).** Possible scores ranged from “0” (no prosocial behavior) to “8” (consistent prosocial behavior).

Children received one point for each prosocial choice made in the resource allocation task. A preliminary analysis of performance on cost versus no cost trials showed no difference between these trials so they were pooled for subsequent analysis (No cost mean = 4.68, SD = 2.02; Cost mean = 4.30, SD = 2.08). For sharing trials, prosocial responses were (1, 1) choices; for envy trials, prosocial responses were choices in which the partner received more than the self. Children thereby received a score ranging from “0” to “8” for each trial type (see **Figure 2** for mean scores).

A 2 × 2 mixed model repeated measures ANOVA with trial type (AI vs. DI) as the within subjects factor, and condition (emotion induction vs. control) as the between subjects factor revealed a significant effect of condition, *F*(1,48) = 4.074, *p* < 0.05, $\eta_{p}^{2}$ = 0.078, with children making overall more prosocial allocations in the emotion induction condition (*M* = 10.00, SD = 3.32) compared to the control (*M* = 7.96, SD = 3.80). A main effect of trial type, *F*(1,48) = 5.995, *p* < 0.05, $\eta_{p}^{2}$ = 0.111 was also observed, with children making more prosocial allocations in AI trials, as opposed to DI trials. No interaction between trial type and condition, *F*(1,48) = 0.062, *p* = 0.804, $\eta_{p}^{2}$ = 0.001, was observed.

Finally, to examine associations among prosocial decisions, personal distress, and empathic concern, bivariate and subsequent partial correlational analyses were conducted. An initial bivariate correlational analysis showed that while there was no relationship between prosocial decisions and emotion ratings for self, *r* = 0.079, *p* = 0.588, there was a significant relation between prosocial decisions and ratings of Jenny’s emotional state, *r* = 0.388, *p* = 0.005. When controlling for rating of Jenny’s emotion there was no relation between overall prosociality and personal distress (emotional ratings for self), *r* = -0.062, *p* = 0.266. However, when controlling for emotion ratings for self, the significant relation between prosociality and empathic concern (ratings of Jenny’s emotional state), *r* = 0.409, *p* = 0.003, remained.

### DISCUSSION

The goal of the current study was to explore experimentally the effects of empathic distress on resource allocation in children. Following work with adults by Barraza and Zak (2009), we predicted that children would exhibit more prosocial behavior toward a protagonist when they were primed by a movie showing the protagonist in distress than when the prime was a neutral movie involving the protagonist. Specifically, we predicted that children in the emotion induction condition (who were primed to experience empathy for their sharing partner) would share more in AI trials, and exhibit less envy in DI trials, thereby showing more generosity in both kinds of trials.

A significant effect of condition in the resource allocation task demonstrated that as hypothesized, children in the emotion induction condition exhibited more prosocial behavior. Children who had been primed with the emotion induction movie shared more in AI trials (more often delivering a benefit), and exhibited less envious behavior in DI trials (less often withholding a benefit), than children in the control condition. Although there was a main effect of trial type with more prosocial behavior in AI trials compared to DI trials, this effect may well reflect the near ceiling response rate in AI trials with no cost to self (the only trial type in which delivering an equitable amount of resources to Jenny was both prosocial, and at no cost to oneself). Significantly, there was no interaction between condition and trial type.

The effect of emotion induction on prosociality appeared to be unaffected by type of decision (AI vs. DI). In other words, the positive effects of the emotion induction on prosocial behavior seems to be consistent across all trial types; having both a positive impact in AI trials – leading to increases in sharing behavior – as well as a neutralizing effect, or negative impact on non-prosocial behavior and consequently producing a decrease in envious behavior in DI trials.

It was important to verify that the specially constructed videos did elicit differences in empathy. Our manipulation check showed that indeed children who watched the emotion induction video reported feeling sadder themselves (evidence of personal distress) and also rated the protagonists emotional state more negatively in comparison to children who viewed the control video (evidence of empathic concern). The relationship between FAS ratings for own emotion, and Jenny’s emotion provide further support that the emotion induction video did elicit empathy, however, the finding that prosociality was correlated with ratings of Jenny’s emotional state, but not with emotional ratings for self suggests that empathic concern more so than personal distress was driving decision making. Despite showing an elevated level of distress after watching the emotion induction video compared to the control video, children’s own level of distress was not significantly related to resource allocation. In contrast, their rating of the protagonist’s distress was. Previous research has also found that personal distress and outward expressions of empathic concern differ in terms of their relation to prosociality – specifically that prosocial intentions and behavior are linked to empathic concern, but not personal distress (e.g., Batson et al., 1981; Eisenberg et al., 1989).

## STUDY 2

The results of study one demonstrated that experiencing empathy for another individual increased subsequent prosocial behavior toward them in children of 5–6 years of age. As a next step, we were interested in exploring whether younger children would show a similar effect. It has been argued that earlier in development, there is a less clear differentiation of personal distress and empathic concern (e.g., Hoffman, 1975, 1982) in situations in which children observe another person in distress. According to Hoffman’s theory, it is around 2–3 years of age that children begin to understand that others have thoughts and feelings different from their own. To explore whether empathy also increases prosocial behavior in younger children, and also whether this potential relationship is linked to personal distress or empathic concern, study one was replicated with 3-year-old children, which is the youngest age for which the task demands of the resource allocation task are appropriate. Pilot testing revealed that 3-year-olds had a difficult time understanding the DI trials, and therefore these trials were excluded.****

### METHOD

#### Participants

Fifty typically developing, 3-year old Canadian children were drawn from a predominately white middle-class neighborhood and randomly assigned to the emotion induction or control conditions. Like the 5–6 year-olds, there were 16 males, and 9 females in each group. The emotion induction group ranged in age from 36 months to 47 months and 28 days (*M* = 43 months, 17 days). The control group ranged in age from 36 months and 1 day to 47 months, 30 days (*M* = 43 months, 10 days).

#### Procedure

The protocol was identical to study one, with one exception. In this study, the DI trials were excluded from the resource allocation task as some younger children struggled with these trial types. Therefore the 3-year-olds participated in a total of eight trials; four AI with cost, and four AI with no cost.

### RESULTS: STUDY 2

#### Manipulation check

**FIGURE 3:**
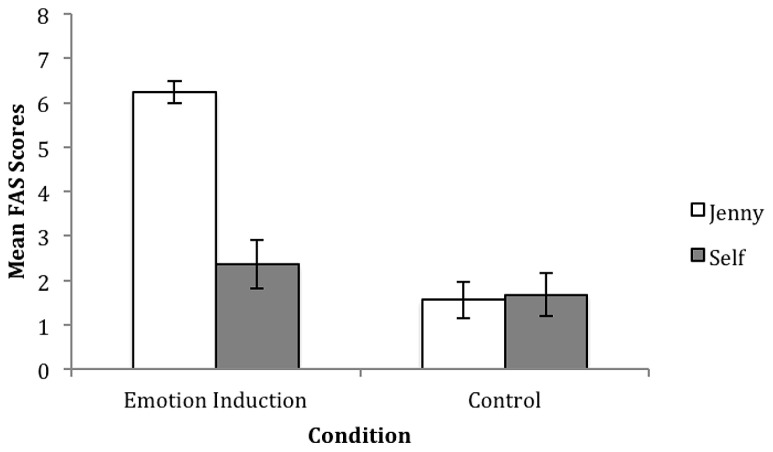
**Mean ratings for Jenny and self on the FAS, with standard error bars, for the emotion induction and control group in study two (3-year-olds).** Possible scores ranged from “0” (very happy) to “8” (very sad).

To assess the effectiveness of the emotion induction video FAS scores for Jenny and self were compared across conditions (see **Figure 3** for mean scores). Independent samples *t*-tests showed that children in the emotion induction condition rated Jenny’s emotion more negatively than children in the control, *t*(48) = 9.464, *p* < 0.01. In contrast to the 5–6 year-olds, no difference between ratings for own emotion was observed, *t*(48) = 0.973, *p* > 0.05. The mean score for Jenny’s emotion was 6.24 (SD = 1.27) in the emotion induction group and 1.56 (SD = 2.12) in the control group, while the mean score for own emotion was 2.36 (SD = 2.77) in the emotion induction group and 1.68 (SD = 2.13) in the control group.

Unlike the older children, a Pearson correlation showed no relationship, *r* = 0.153, *p* > 0.05, between emotion ratings for Jenny and self.

#### Main analysis

**FIGURE 4:**
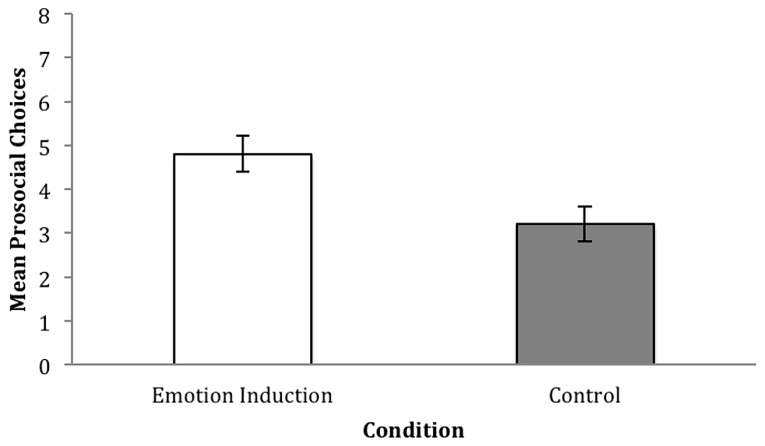
**Mean prosocial choices on the resource allocation task with standard error bars, for the emotion induction, and control group, in AI trials in study two (3-year-olds).** Scores in cost and no cost trials were pooled into one overall prosocial score, and possible scores ranged from “0” (no prosocial behavior) to “8” (consistent prosocial behavior).

Children received one point for each prosocial choice made in the resource allocation task (1, 1 in both AI trials). Children thereby received a score ranging from “0” to “4” for each trial type, and an overall prosocial score ranging from “0” to “8” (see **Figure 4** for mean scores).

A 2 × 2 mixed model repeated measures ANOVA with trial type (cost vs. no cost) as the within subjects factor, and condition (emotion induction vs. control) as the between subjects factor revealed a significant effect of condition, *F*(1,48) = 6.869, *p* < 0.05, $\eta_{p}^{2}$ = 0.125, with children making overall more prosocial allocations in the emotion induction condition (*M* = 4.8, SD = 2.06) compared to the control (*M* = 3.2, SD = 2.0). A main effect of cost, *F*(1,48) = 34.505, *p* < 0.01, $\eta_{p}^{2}$ = 0.418 was also observed, with children making more prosocial allocations in no cost trials (*M* = 2.5, SD = 1.31), as opposed to cost trials (*M* = 1.5, SD = 1.2). No interaction between cost and condition, *F*(1,48) = 0.129, *p* > 0.05, $\eta_{p}^{2}$ = 0.003, was observed.

Finally, correlations between prosocial decisions, and emotion ratings for self, as well as Jenny, were conducted. In contrast to the older children there was no strong relation between overall prosociality and emotion ratings for self, *r* = 0.074, *p* = 0.609, or Jenny, *r* = 0.179, *p* = 0.215. Further, no relationships were observed between self-reported emotion and prosociality when controlling for ratings of Jenny’s emotion, *r* = 0.048, *p* = 0.742 or between prosociality and ratings of Jenny’s emotional state, *r* = 0.170, *p* = 0.244 when controlling for rating’s of one’s own emotion.

### DISCUSSION

The purpose of study two was to explore whether the positive effects of empathy on prosociality extended to a younger age group, and whether the effects were more closely tied to personal distress or the empathic concern component of empathy. It was hypothesized that empathy would increase prosociality in 3-year-olds, as it did with 5–6 year-olds, but what was of particular interest was whether personal distress would be a stronger influence in younger children, who may be less able to distinguish their own emotions from those of another individual in distress. The method from study one was slightly modified to accommodate the younger children, as the DI trials were found to be difficult for them to understand, and were therefore excluded.

Consistent with study one, an effect of condition was observed with 3-year-olds making more prosocial allocations in comparison to children in the control group. This finding supports the hypothesis that empathy leads to increased prosocial behavior in young, 3-year-old children (at least in AI trials) in addition to older, school aged children.

Explorations of how 3-year-olds rated Jenny’s emotion showed that our experimental manipulation produced group differences in empathic concern, as children in the emotion induction condition rated Jenny as feeling sadder than children in the control. However, no differences in self-rated emotion were found between groups. It could be the case that younger children are just not as skilled at recognizing or articulating how they themselves feel in response to witnessing another in a distressing situation, which is perhaps the most likely explanation. These difficulties in using self-report measures with young children have been recognized in the literature (Eisenberg and Fabes, 1990). Difficulty comprehending self-report questions, as well as accurately identifying one’s own emotional state, and differentiating between closely related emotional states, have been identified as concerns to be aware of with this population. However, it could simply be the case that our manipulation was not successful in inducing personal distress in younger children.

Finally, correlational analyses showed that prosociality was correlated with neither ratings of Jenny’s emotion nor ratings of own emotion. One potential contributor to this discrepancy with findings for the older children could be a lack of power, as 5–6 year olds participated in double the number of trials (both AI and DI, as opposed to AI alone). Alternatively, if younger children are less able to accurately identify, or verbalize their own, and others’ emotions as previously suggested, this inability could also be contributing to the null finding. Including a measure of facial distress would be useful to include in subsequent research with this age group, to more accurately gage personal distress if it is suspected that 3-year-old children are too young to accurately express their own emotions.

## GENERAL DISCUSSION

The current studies explored the relationship between empathy and prosocial behavior in children. It was hypothesized that experiencing empathy toward one’s partner would both increase prosocial behavior, and decrease non-prosocial behavior. As hypothesized, both 5–6 year-olds, and 3-year-olds showed increased prosocial behavior, and 5–6 year-olds showed decreased non-prosocial behavior toward their partner, if they had first been primed to feel empathy for them. It is important to note that the induced emotion in these experiments was negative, and more specifically, sadness. Empathic experiences of other emotions, or psychological, or physical states in others, such as happiness, fear, pain, etc., may not influence prosociality in the same way, although it is worth exploring how empathic experiences of other negative emotions or states, as well as positive emotions or states, influence prosociality.

As the stimulus videos were created for the purpose of this research, it was important to validate their effectiveness. The fact that children in both studies rated the character as sadder after watching the emotion inducing video than after watching the neutral video provides important validation for the emotion induction manipulation.

Also of interest was whether personal distress or empathic concern could be specifically linked to increases in prosociality. Though the condition effect was consistent across age groups, differences in self-reports of own emotion, and the relationship between prosociality and empathic concern differed between studies one and two. Specifically, in study one, group differences were observed for both personal distress, and empathic concern, and prosociality was correlated with empathic concern (but not personal distress) in 5–6 year-old children. This finding is in line with previous research (e.g., Batson et al., 1981; Eisenberg et al., 1989) suggesting that an outward orientation of empathic concern is related to prosociality, whereas personal distress is not.

In contrast, in experiment two there was no group difference observed in self-rated emotions, and neither personal distress or empathic concern were correlated with prosocial behavior for the 3-year-olds. These differences across age groups could reflect the inability of younger children to accurately reflect on their own emotion, as well as the methodological differences between experiments. As children behaved differently following exposure to the emotion induction vs. control video, and the videos produced group differences in reports of both personal distress and empathic concern – with the exception of personal distress in 3-year-olds – we feel confident that the videos were effective in inducing empathy in both experiments.

Overall, our experiments support the findings of Barraza and Zak (2009) that experiencing empathy for sadness leads to more prosocial behavior, and extends this finding to children across two distinct age groups. Although similar in concept, it is important to note that our studies differ from Barraza and Zak’s (2009) study in a number of ways. First, our videos were closely matched across conditions. Participants both saw a little girl named Jenny playing with her dog, making posters, and hanging them around her neighborhood. They heard her narrate the video, which was matched for factors such as word count, and length. The primary difference between videos was the negative emotion Jenny portrayed in the emotion induction video. Further, the use of the Resource Allocation task allowed for multiple trials, and an exploration of the effects of empathy on both AI and DI trial types so the potential of empathy to reduce non-prosocial behavior could also be examined in 5–6 year-olds. Finally, in this study the partner with whom participants shared was the individual toward whom they were primed to feel empathy, as opposed to an unrelated partner. Whether empathic concern for sadness toward one person would lead children to behave more prosocially with an unrelated partner is unknown at this point and is a question for future research.

It may be noted that our measures of empathy were both self-report and so might be open to concerns about validity. However, similar (verbal) self-report approaches have been commonly used in related research (e.g., Feshbach and Roe, 1968; Eisenberg et al., 1996; Strayer and Roberts, 1997). Importantly, we found that 5–6 year old children’s attribution of emotion to a partner in a distressing situation predicted sharing behavior with this individual, thereby providing some validation of the usefulness of this self-report measure.

In both experiments, children were first asked to identify how Jenny felt, and then to express how they themselves felt. As ratings of Jenny’s emotion were obtained first, this measure was unaffected by how children may have felt themselves. Recall ratings of Jenny’s emotion differed across groups in both experiments, and were correlated with prosociality in experiment 1. Ratings for participants’ own emotion were collected subsequently, allowing all children to first reflect on how Jenny felt before communicating their own emotional state. These ratings of own emotion were not correlated with prosociality, and did not differ between groups in experiment two. Although it is unlikely that the order in which the questions were asked influenced the results (especially since it would be the second question influenced by the first which does not seem to be the case), it is worth mentioning that further explorations may benefit from counterbalancing the order of these two questions.

Although the relation between empathy and sympathy and prosocial behavior has been explored in earlier work (e.g., Eisenberg et al., 1988; Zahn-Waxler and Radke-Yarrow, 1990), this is the first experimental demonstration to our knowledge of empathy for sadness, and specifically empathic concern being shown to influence resource allocation in young children. Furthermore, our results suggest empathic concern for sadness can promote sharing, but perhaps the most novel contribution of this work is the finding that it also has a counteracting, or neutralizing effect on the negative consequences of envy.

In sum, these experiments show that empathic concern for sadness does lead to prosocial resource allocation in young children both by promoting sharing and decreasing envy. Understanding the development of prosocial behavior is important in many regards. Prosocial development is both important in creating and sustaining personal relationships, and on a larger scale, a critical component in maintaining a functioning society. By understanding the mechanisms such as empathy, that influence prosocial behavior, we can better support and encourage the development of prosocial behaviors such as sharing, and learn how to inhibit or neutralize more negative aspects of social behavior such as envy.

## Conflict of Interest Statement

The authors declare that the research was conducted in the absence of any commercial or financial relationships that could be construed as a potential conflict of interest.
